# Genome Sequence of *Bacillus cereus* Strain LCT-BC235, Carried by the Shenzhou VIII Spacecraft

**DOI:** 10.1128/genomeA.00665-13

**Published:** 2014-01-09

**Authors:** Longxiang Su, Tong Wang, Lisha Zhou, Chunyan Wu, Yinghua Guo, De Chang, Yan Liu, Xuege Jiang, Sanjun Yin, Changting Liu

**Affiliations:** Nanlou Respiratory Diseases Department, Chinese PLA General Hospital, Beijing, Chinaa; Medical College, Nankai University, Tianjin, Chinab; BGI-Shenzhen, Shenzhen, Chinac

## Abstract

In order to explore the effect of space environments on *Bacillus cereus*, we determined the draft genome sequence of a *B. cereus* strain, LCT-BC235, which was isolated after space flight.

## GENOME ANNOUNCEMENT

Many previous studies have confirmed that the space environment can have a strong effect on a variety of microbial properties, such as antibiotic efﬁcacy, microbial virulence, and biochemical metabolism (1, 2). *Bacillus cereus* is one of the most common bacteria that may be a threat to human health or harmful to the structural materials of spacecraft and space stations (3). *B. cereus* strain LCT-BC235 exhibited a low growth rate, resistance to ceftazidime, and changes in metabolism compared to the ground control strain LCT-BC244.

After extraction of the genomic DNA of *B. cereus* LCT-BC235, whole-genome sequencing was performed using IlluminaHiSeq 2000 (Illumina, Inc.) by generating paired-end libraries with an insert size of 350 bp and mate-paired libraries with an insert size of 6,000 bp. The read length was set to 90 bp. After filtering of the low-quality data, 580 Mb and 246 Mb of data were generated separately. Then, we assembled the short reads into genome sequence using SOAPdenovo v 1.05, and the scaffolds were manually connected according to paired-end relationships. The gaps were filled using GapCloser with read mapping information. Finally, we obtained 5 scaffolds consisting of 34 contigs with a total length of 5,148,728 bp and determined the G+C content to be 35.32%. The coding sequences (CDS) were predicted by using Glimmer v 3.02. From the genome analysis results, we found that the genome contained 5,258 genes with an average length of 836 bp, and the total length of genes was 4,395,270 bp, which makes up 85.37% of the genome. Homologous comparison by BLAST was performed for all of the genes with the NCBI nonredundant public database and other databases. There were 2,765 CDS involving the 22 functional COG groups and a part of the CDS involving the 34 metabolic pathway KEGG groups.

According to RNAmmer and tRNAScan-SE, a total of 59 noncoding RNAs were found in the genome, including 53 tRNA, 2 rRNAs, and 4 sRNA. In addition, 256 minisatellite DNAs and 20 microsatellite DNAs were determined based on the Repbase transposable elements library or using RepeatMasker and Repeat-ProteinMasker. Furthermore, 334 tandem repeats were predicted using TRF software.

### Nucleotide sequence accession number.

This whole-genome shotgun project of *Bacillus cereus* LCT-BC235 has been deposited at DDBJ/EMBL/GenBank under the accession number ATHN00000000. The version described in this paper is the first version.
